# Metabolic Hydrolysis of Aromatic Amides in Selected Rat, Minipig, and Human *In Vitro* Systems

**DOI:** 10.1038/s41598-018-20464-4

**Published:** 2018-02-05

**Authors:** Peter R. Bradshaw, Ian D. Wilson, Rachel Upcott Gill, Philip J. Butler, Clive Dilworth, Toby J. Athersuch

**Affiliations:** 1 Department of Surgery and Cancer, Faculty of Medicine, Imperial College London, Exhibition Road, South Kensington, London, SW7 2AZ UK; 2 Cyprotex, Alderley Park, Nether Alderley, Cheshire, SK10 4TG UK; 3 MRC-PHE Centre for Environment and Health, School of Public Health, Imperial College London, Norfolk Place, London, W2 1PG UK

## Abstract

The release of aromatic amines from drugs and other xenobiotics resulting from the hydrolysis of metabolically labile amide bonds presents a safety risk through several mechanisms, including geno-, hepato- and nephrotoxicity. Whilst multiple *in vitro* systems used for studying metabolic stability display serine hydrolase activity, responsible for the hydrolysis of amide bonds, they vary in their efficiency and selectivity. Using a range of amide-containing probe compounds (0.5–10 µM), we have investigated the hydrolytic activity of several rat, minipig and human-derived *in vitro* systems - including Supersomes, microsomes, S9 fractions and hepatocytes - with respect to their previously observed human *in vivo* metabolism. In our hands, human carboxylesterase Supersomes and rat S9 fractions systems showed relatively poor prediction of human *in vivo* metabolism. Rat S9 fractions, which are commonly utilised in the Ames test to assess mutagenicity, may be limited in the detection of genotoxic metabolites from aromatic amides due to their poor concordance with human *in vivo* amide hydrolysis. In this study, human liver microsomes and minipig subcellular fractions provided more representative models of human *in vivo* hydrolytic metabolism of the aromatic amide compounds tested.

## Introduction

Exposure to aromatic amines (AA) is of concern in pharmaceutical development, as well as in occupational and wider environmental contexts, due to their potential toxicities; some AA structures have been shown to be responsible for genotoxicity^1^, hepatotoxicity^2^ and the induction of methaemoglobin formation^3^. AA substructures are found in a wide range of organic compounds, commonly incorporated via amide bonds. Appropriate process control can minimise/eliminate AA impurities in a drug formulation, but cannot address the potential for *in vivo* metabolic hydrolysis that may liberate amines as metabolites. Upon release, AA are often metabolically bioactivated, and formation of *N*-hydroxylamine metabolites has been proposed as a mechanism of toxicity, especially in the case of genotoxicity^4^. Failure to detect the presence of toxic AA produced as metabolites from candidate drugs early in drug development can result in costly late attrition.

Several widely-used therapeutic drugs and drug candidates have displayed *in vivo* hydrolytic release of AA, alongside concomitant toxicity, whilst close structural analogues have not. A good example of this is given by flutamide, an antiandrogen used in the treatment of prostatic carcinoma, which undergoes substantial metabolism *in vivo* with hydrolysis of the amide bond liberating 3-trifluoromethyl-4-nitroaniline. This aniline has been detected in the plasma of patients after oral administration of the drug^5^ and exposure has been associated with hepatotoxicity^2^. By contrast, the structurally-related antiandrogen bicalutamide, does not undergo amide hydrolysis in man^6^, with few cases of hepatotoxicity having been reported in the literature^7^. The aniline analgesics also display varied metabolism; paracetamol (acetaminophen) undergoes limited hydrolysis to the amine, *p*-aminophenol after administration to humans with only ca. 1–2% of the dose being hydrolysed^8^, whereas acetanilide undergoes hydrolysis at a higher rate with approximately 10% of the dose hydrolysed^9,10^. The anaesthetics prilocaine and lidocaine along with the herbicide propanil also undergo hydrolysis in humans^11–13^ and exposure to their amine metabolites has been shown to induce methaemoglobinemia *in vitro* and in patients^3,13,14^. In contrast niclosamide, used to treat cestode infections, has been shown to be stable to hydrolysis *in vivo*^15^.

**Figure 1:**
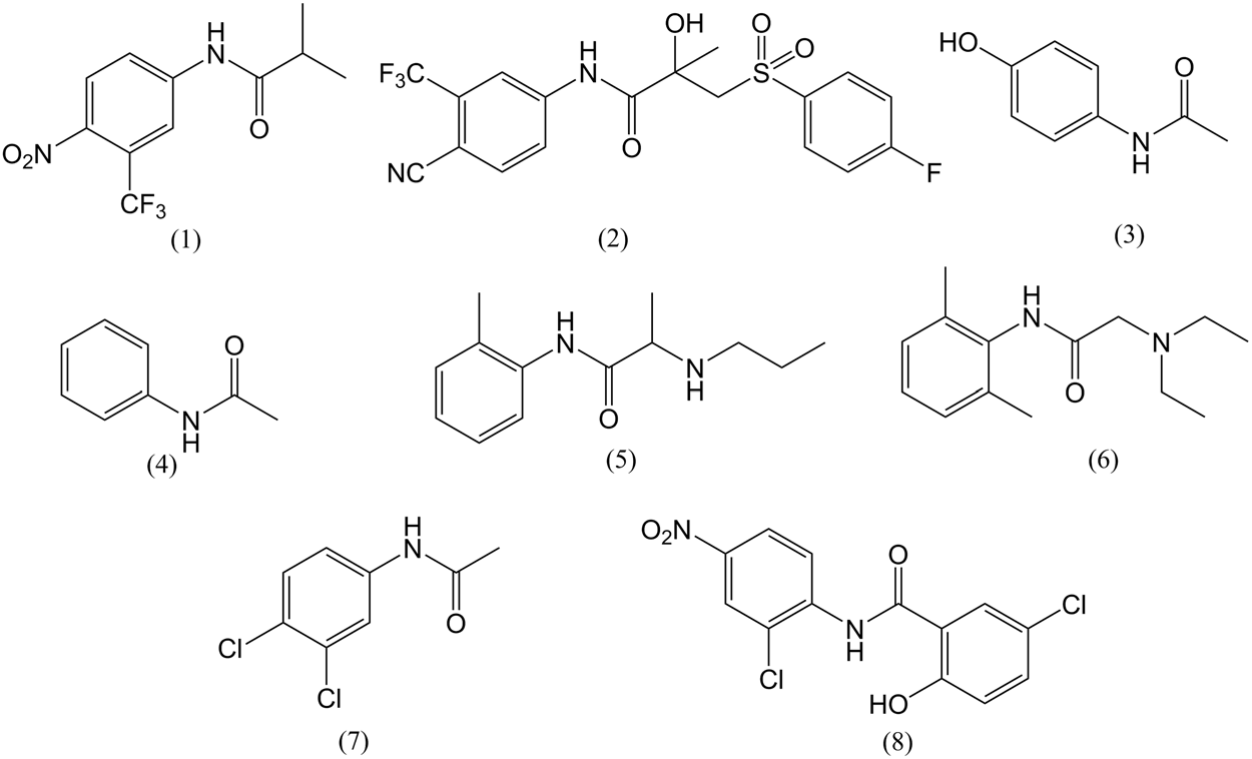
Chemical structures of probe aromatic amides with well-characterised *in vivo* metabolism, used for the comparison of *in vitro* systems. (1) Flutamide (2) Bicalutamide (3) Paracetamol (4) Acetanilide (5) Prilocaine (6) Lidocaine (7) Propanil and (8) Niclosamide.

One challenge for scientists working in the area of drug discovery is accurately and rapidly predicting the metabolic susceptibility of aniline-containing candidate drugs to the actions of serine hydrolases, the main enzymes responsible for amide hydrolysis. Key members of this family include the membrane bound carboxylesterases CES1 and CES2 which are also present in the cytosol, along with arylacetamide deacetylase AADAC which is only found within the endoplasmic reticulum^16,17^. CES and AADAC are expressed in the liver and small intestines; CES1 is the predominant isoform expressed in the liver and has relatively low expression in the small intestines, where CES2 is the main isoform^16,18^. Prior to human exposure careful selection of predictive *in vitro* and animals models which are representative of human metabolism are essential. Whilst there are multiple *in vitro* systems available from different species which show hydrolase activity, not all are equally efficient or demonstrate similar substrate selectivity. Recently, Jones *et al*.^19^ performed retrospective analysis of the metabolism of 38 development compounds in minipig *in vitro* systems compared with similar systems derived from human, rat, dog, monkey, rabbit and mouse. Minipig generally showed good correlation with human *in vitro* metabolism, however metabolite coverage of minipig incubations differed from human in a small number of compounds due to amide hydrolysis^19^. Comparison of species with respect to amide hydrolysis of commercially available compounds demonstrated a significantly higher activity for the minipig S9 fractions compared to human S9 preparations for this type of biotransformation. Rat S9 fractions showed similar rates of hydrolysis compared to human but differed in the number of amides hydrolysed^19^. Here we report the amide hydrolytic activity of multiple *in vitro* systems originating from rat, minipig and human. We assessed the ability of these *in vitro* systems to predict the *in vivo* hydrolysis of eight aromatic amide-containing compounds (Fig. 1) for which the human *in vivo* metabolic fate of the amide bond had previously been characterised, alongside 15 exemplar aromatic amides.

## Results

### Selection of compounds

The initial selection criteria for compounds was the presence of an formanilide substructure, that would yield an aromatic amine metabolite if hydrolysed. In order to compare *in vitro* to *in vivo* metabolism eight amides were chosen where the *in vivo* metabolism in humans had already been well characterised (Fig. 1). An additional set of aromatic amides with unknown *in vivo* metabolism were selected using a systematic approach to investigate substituent effects of e.g., electron withdrawing (-NO_2_, -F) and electron donating groups (-OH, -CH_3_) which were varied between compounds around the formanilide ring (*ortho*-, *meta*- and *para*- to the amide bond) for future quantitative structure-activity relationship (QSAR) modelling. All 23 compounds were assayed in each of the *in vitro* systems described below.

### Hydrolysis of amides by human carboxylesterase-containing Supersomes

**Figure 2:**
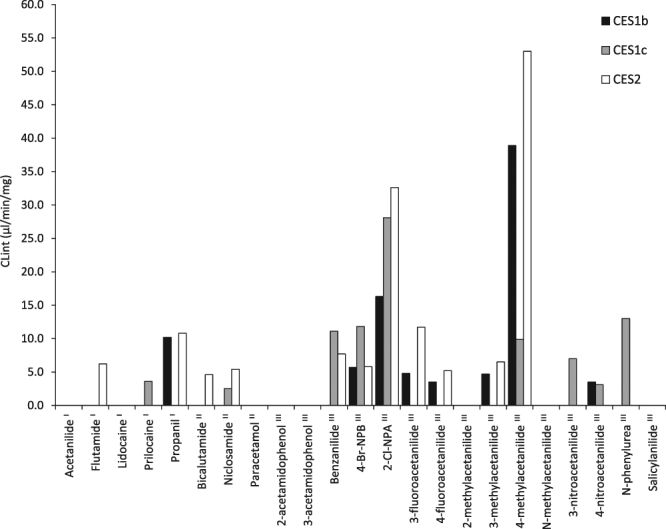
Hydrolysis of amides by human carboxylesterase Supersomes. Compounds labelled ^I^ are hydrolysed in humans, ^II^ are not hydrolysed in humans and ^III^ have unknown human *in vivo* metabolism. 4-Br-NPB and 2-Cl-NPA represent 4-bromo-2-methoxy-*N*-phenylbenzamide and 2-chloro-*N*-phenylacetamide.

Supersomes - recombinant enzymes expressed in vesicles^20^ - allow the direct study of action of individual enzymes. Three carboxylesterase isoforms CES1b, CES1c and CES2 were investigated using Supersomes (AADAC Supersomes were commercially unavailable) as shown in Fig. 2. The hydrolysis of amides by the recombinant enzymes differed from human *in vivo* metabolism; bicalutamide and niclosamide hydrolysis has not been observed *in vivo*, however hydrolysis of bicalutamide occurred within CES2 incubations and hydrolysis of niclosamide was observed in CES1c and CES2 incubations. In contrast, hydrolysis of acetanilide and lidocaine has been observed *in vivo* previously but was not observed in the Supersome incubations.

### Amide metabolism by human subcellular fractions

**Figure 3:**
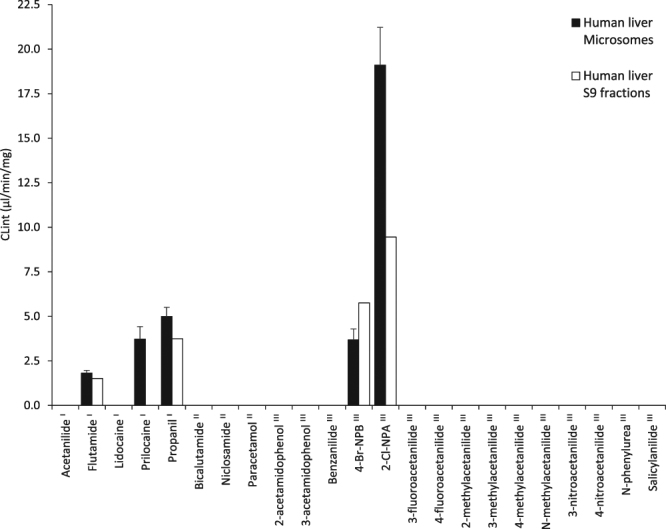
Hydrolysis of amides by human liver microsomes and S9 fractions. Compounds labelled ^I^ are hydrolysed in humans, ^II^ are not hydrolysed in humans and ^III^ have unknown human *in vivo* metabolism. 4-Br-NPB and 2-Cl-NPA represent 4-bromo-2-methoxy-*N*-phenylbenzamide and 2-chloro-*N*-phenylacetamide.

Hepatic microsomes and S9 fractions obtained from differential centrifugation of homogenised liver^21^ contain a full complement of CES1, CES2 and AADAC enabling the action of carboxylesterases and AADAC to be studied simultaneously. The series of amides were assayed with human liver microsomes and S9 fractions (Fig. 3). Overall, human subcellular fractions showed a narrower substrate specificity compared to Supersomes, with microsomes hydrolysing five of the 23 amides assayed and S9 fractions hydrolysing only four. In contrast to Supersomes, microsomes and S9 fractions provided a more accurate model of *in vivo* metabolism as the hydrolysis of bicalutamide and niclosamide seen with Supersomes was absent. However, like Supersomes, hydrolysis of acetanilide and lidocaine was not observed in incubations with either system.

### Amide metabolism by rat S9 fractions

**Figure 4:**
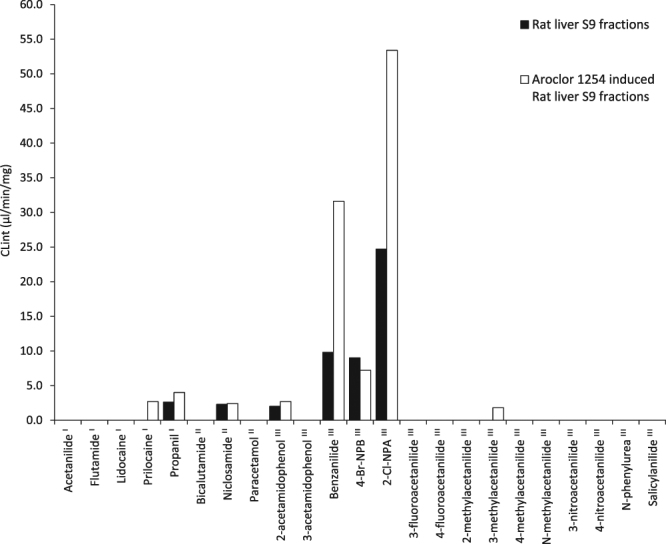
Hydrolysis of amides by rat liver S9 fractions and Aroclor 1254 induced S9 fractions. Compounds labelled ^I^ are hydrolysed in humans, ^II^ are not hydrolysed in humans and ^III^ have unknown human *in vivo* metabolism. 4-Br-NPB and 2-Cl-NPA represent 4-bromo-2-methoxy-*N*-phenylbenzamide and 2-chloro-*N*-phenylacetamide.

Rat S9 metabolic systems are commonly used in the bacterial reverse mutation assay (Ames test) to assess the mutagenicity of compounds^22^. Aroclor 1254 induced rat metabolic systems are regularly utilised in Ames tests and are required by regulators in the drug development process; the induction process results in a large increase in the enzyme activity of multiple CYPs including the CYP1A subfamily^23^. The effect of Aroclor 1254 induction on amide hydrolytic capacity of rat liver S9 was investigated (Fig. 4). Of the eight compounds with known *in vivo* metabolism incubated with Aroclor 1254-naïve rat S9, four remained unmetabolised (acetanilide, flutamide, lidocaine or prilocaine). Two main differences in the pattern of metabolism were observed as a result of Aroclor 1254 induction; hydrolysis of two additional compounds (prilocaine and 3-methylacetanilide), and enhanced rate of hydrolysis of two others (benzanilide and 2-chloro-*N*-phenylacetamide). Importantly, the induction of prilocaine hydrolysis provides a closer match to the known *in vivo* fate in man. The hydrolysis of acetanilide, flutamide and lidocaine was absent from the Aroclor 1254 induced rat incubations. Flutamide undergoes amide hydrolysis following administration to humans^5^ but this was not recapitulated in either rat S9 incubation. Previous *in vitro* studies have demonstrated hydrolysis of flutamide mainly occurs by the action of AADAC^24^ and hydrolysis has also been observed within rat liver microsomes^25^. However, the expression of AADAC in rat tissue is seven-fold lower than the expression in human tissue^18^ which may account for the absence of observable flutamide hydrolysis in this rat S9 model.

### Amide metabolism by minipig subcellular fractions

**Figure 5:**
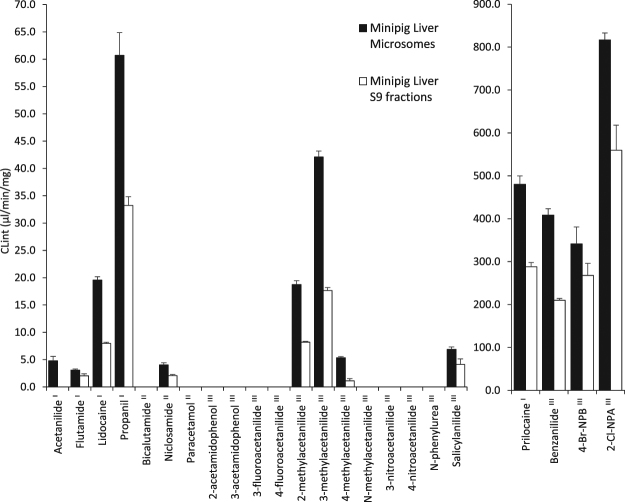
Hydrolysis of amides in minipig liver microsomes and S9 fractions. Compounds labelled ^I^ are hydrolysed in humans, ^II^ are not hydrolysed in humans and ^III^ have unknown human *in vivo* metabolism. 4-Br-NPB and 2-Cl-NPA represent 4-bromo-2-methoxy-*N*-phenylbenzamide and 2-chloro-*N*-phenylacetamide.

Following the poor concordance of rat *in vitro* systems to human *in vivo* metabolism, the metabolic fate of the series of amides was determined in Gottingen minipig microsomes and S9 fractions (Fig. 5). Utilisation of minipig *in vitro* systems has gained in popularity due to the increasing use of minipig as a non-clinical test species in drug development. In our hands, minipig microsomes demonstrated a close representation of human *in vivo* metabolism with the successful hydrolysis of acetanilide, flutamide, lidocaine, prilocaine and propanil (Fig. 5). Minipig S9 preparations differed from minipig microsomes only in the case of acetanilide which was not hydrolysed by the S9 fractions. Hydrolysis of acetanilide was absent in all of the rat and human models discussed above, despite hydrolysis being observation *in vivo* at ca. 10% of the administrated dose^9,10^. Detection of the hydrolysis of acetanilide by minipig microsomes demonstrates sensitivity to amides which are hydrolysed at low levels in humans *in vivo*. Hydrolysis of paracetamol was not observed in any (rat, minipig or human) of the *in vitro* systems assessed. This is not unexpected due to the very low hydrolysis of paracetamol observed in humans (ca. 1–2%)^8^. Minipig subcellular fractions differed from human *in vivo* metabolism in the case of niclosamide (the hydrolysis of niclosamide was also observed in Supersomes and rat S9 fractions). These results may represent an elevated amide hydrolysis activity for minipig compared to human *in vivo* metabolism, as has previously been reported^19^. In the present study, very rapid hydrolytic activity in the minipig incubations was observed for four amides (Fig. 5).

### Amide hydrolysis by human hepatocyte incubations

In addition to the subcellular systems from rat, minipig and human, the series of amides were assayed with cryopreserved primary human hepatocytes, which are often obtained from liver resection patients and more closely represent *in vivo* conditions^11^. Their increased complexity and metabolic capacity increases the difficulty of examining individual metabolic reactions in isolation (e.g. amide hydrolysis). Following incubation with hepatocytes, amine metabolites were detected in four of the 23 amide incubations (bicalutamide, flutamide, lidocaine and prilocaine), indicative of hydrolysis. Unlike the other human- and rat-derived systems, hydrolysis of lidocaine was observed in these hepatocyte incubations. Previous studies, in rat and rabbit liver slices have documented that lidocaine initially undergoes *N*-dealkylation prior to amide hydrolysis^26^; *N*-dealkylation of lidocaine produces monoethylglycinexylidide that undergoes amide hydrolysis more readily than the parent. CYP3A4 has been demonstrated to be responsible for the *N*-dealkylation of lidocaine *in vitro*^27^ and this oxidation reaction requires NADPH as a co-factor. In the subcellular incubations performed in this study NADPH was excluded to allow the direct study of the activity of serine hydrolases. By contrast, hepatocyte incubations are not constrained by a lack of NADPH which may have resulted in *N*-dealkylation, thereby assisting the amide hydrolysis of lidocaine. Minipig subcellular fractions hydrolysed lidocaine without requiring *N*-dealkylation suggesting minipig serine hydrolases have a greater capacity for amide hydrolysis than rat and human hydrolases.

## Discussion

Hydrolysis of aromatic amide-containing compounds *in vivo* can lead to toxicity due to the release of aromatic amine metabolites and therefore the avoidance, or identification of, potentially problematic aromatic amide structures is clearly valuable. We assayed eight amides in a range of *in vitro* systems to identify those which showed the greatest concordance with human *in vivo* hydrolytic metabolism. These studies revealed a wide range in performance of the *in vitro* systems, illustrating the variation in substrate specificity of serine hydrolases from different species. Comparison of the human *in vitro* hydrolysis systems demonstrated clear differences between the activity of serine hydrolase between human-derived tissue preparations and the recombinant enzymes. Collectively the carboxylesterase Supersomes performed the least well, with their metabolism matching human *in vivo* metabolism in the case of only four out of eight amides. Compared to Supersomes, human S9 and hepatocyte-based assays showed slightly improved metabolite coverage with human liver microsomes providing the closest representation of human *in vivo* amide hydrolysis (differing from *in vivo* metabolism in the cases of acetanilide, paracetamol and lidocaine).

Rat S9 fractions are commonly used in the Ames test to assess the mutagenicity of compounds which require metabolic activation. In our hands, rat S9 preparations demonstrated limited concordance with human *in vivo* amide hydrolysis, with only a modest improvement resulting from induction by Aroclor 1254. The utility of using rat S9 in a critical assay for detecting genotoxicity may be limited by their minimal aromatic amide hydrolysis ability. Unlike rat-derived systems, minipig subcellular fractions strongly resembled human *in vivo* metabolism in terms of amide hydrolysis based on the compounds assayed in this study. We have observed that minipig *in vitro* systems provide a close representation of human *in vivo* metabolism based on the compounds investigated in the current study. Notwithstanding the likely differences between the substrate specificity between different species, our findings support the consideration of minipig systems as a complementary assay to be used in conjunction with the traditional Ames test. This may better aid the prediction/detection of human-relevant genotoxic aromatic amine metabolite release at an earlier stage in drug development, and help limit late candidate attrition. In summary, the hydrolytic activity of multiple *in vitro* systems was assessed using a range of aromatic amides, the results show a clear deficiency in that ability of several human and rat-derived *in vitro* assays to detect human-relevant metabolites. However, the close resemblance of human *in vivo* metabolism demonstrated by minipig systems clearly warrants further investigation with additional aromatic amide-containing compounds.

## Methods

### Materials

Reagents were ≥95% purity and were used as provided without further purification or characterisation. 2-chloro-*N*-phenylacetamide, 4-bromo-2-methoxy-*N*-phenylbenzamide, 3-fluoroacetanilide, *N*-methylacetanilide and 4-nitroacetanilide were purchased from Fluorochem (Hadfield, UK). 2-Acetamidophenol, 3-acetamidophenol, acetanilide, acetonitrile, ammonium acetate, ammonium formate, benzanilide, dimethyl sulphoxide (DMSO), formic acid, paracetamol (acetaminophen) and salicylanilide were purchased from Thermo Fisher Scientific (Hemel Hempstead, UK). Bicalutamide, flutamide, L-glutamine, 4-(2-hydroxyethyl)piperazine-1-ethanesulphonic acid (HEPES), lidocaine hydrochloride, 3-methylacetanilide, 4-methylacetanilide, niclosamide, *N*-phenylurea, potassium dihydrogen phosphate, propanil, sodium phosphate dibasic, Trypan Blue and Williams’ Medium E were purchased from Sigma Aldrich (Gillingham, UK). 4-Fluoroacetanilide, 2-methylacetanilide, 3-nitroacetanilide and prilocaine hydrochloride were purchased from VWR International Ltd (Lutterworth, UK).

### Enzyme sources

Pooled subcellular fractions of male Sprague-Dawley rat liver S9 fractions (10 donors), male Sprague-Dawley Aroclor 1254 induced rat liver S9 fractions (20 donors), human liver S9 fractions (mixed gender 10 donors), male Gottingen minipig liver microsomes (3 donors) and male Gottingen minipig liver S9 fractions (3 donors) were purchased from BioreclamationIVT (West Sussex, UK). Cryopreserved primary human hepatocytes (mixed gender 10 donors) were also purchased from BioreclamationIVT. Human CES1b, CES1c and CES2 Supersomes and human liver microsomes (mixed gender 150 donors) were purchased from Corning (Flintshire, UK).

### Subcellular incubations

Each of the 23 aromatic amide test compounds (bicalutamide, niclosamide, flutamide, lidocaine, prilocaine, propanil, acetanilide, 2-acetamidophenol, 3-acetamidophenol, acetaminophen, 3-nitroacetanilide, 4-nitroacetanilide, 3-fluoroacetanilide, 4-fluoroacetanilide, 2-methylacetanilide, 3-methylacetanilide, 4-methylacetanilide, *N*-methylacetanilide, benzanilide, salicylanilide, 4-bromo-2-methoxy-*N*-phenylbenzamide, *N*-phenylurea, 2-chloro-*N*-phenylacetamide) were dissolved in DMSO at a concentration of 10 mM and stored at −20 °C until required (maximum storage time three months). *In vitro* incubations were prepared with Supersomes (CES1b, CES1c and CES2), microsomes or S9 fractions (human, rat and minipig) in 0.1 M phosphate buffer pH 7.4 at 1 mg/ml protein concentration to encourage turnover of the amide aiding the detection of aromatic amine metabolites and confirmation of hydrolysis. Reaction mixtures were pre-incubated at 37 °C with shaking for 10 minutes using an Eppendorf ThermoMixer Comfort (Stevenage, UK). Incubations were initiated by the addition of test amide (final concentration 0.5 µM, 0.25% DMSO) and baseline (t_0_) samples were taken immediately and quenched in ice cold acetonitrile with 1% formic acid. Vehicle control incubations were initiated with equivalent amount of DMSO. Samples were acquired from the supersome incubations at 5, 15, 30 and 45 minutes and quenched in acetonitrile containing 1% (v/v) formic acid. Incubations with microsomes and S9 fractions were sampled at 15, 30, 60 and 120 minutes. Additional 5 minute time points were acquired for the minipig assays due to the rapid hydrolysis observed with several amides. Quenched plates were centrifuged at 1328 × g for 30 minutes at 4 °C. Supernatants were transferred to clean sample plates and diluted 1:1 with 0.55 µM metoprolol (internal standard) in water for liquid chromatography-tandem mass spectrometry (LC-MS/MS) analysis. Following initial screening with a single replicate, human liver microsomes, minipig microsomes and minipig S9 fraction assays were repeated in triplicate.

### Hepatocyte incubations

Cryopreserved human hepatocytes were thawed and diluted in fresh modified William’s E media pH 7.4 (supplemented with 2mM L-glutamine and 25 mM HEPES). Cells were pelleted by centrifugation (50 × g for 5 minutes at 22 °C) re-suspended in fresh media and the cell viability was determined by Trypan Blue exclusion. Hepatocyte incubations were prepared at a cell density of 0.5 × 10^6^ cells in media and were pre-incubated for 15 minutes at 37 °C with shaking using an Eppendorf ThermoMixer Comfort. Reactions were initiated by the addition of test amide (final concentration 10 µM, 0.25% DMSO) and baseline (t_0_) samples were taken immediately and quenched in ice cold acetonitrile containing 1% (v/v) formic acid. Vehicle control incubations were initiated with equivalent amount of DMSO. Samples were acquired at 10, 20, 40, 60 and 120 minutes and quenched. Quenched reactions were spun at 1328 × g for 30 minutes at 4 °C. Supernatants were transferred to clean sample plates, diluted with 0.55 µM metoprolol in water and were analysed by LC-MS/MS for amine metabolites.

### LC-MS/MS instrumentation and conditions

LC-MS/MS analysis was performed using a Waters UPLC instrument with an Acquity^TM^ binary solvent manager, Acquity^TM^ 4-position heated column manager and 2777 Ultra High Pressure Autosampler coupled to a Xevo-TQ MS triple quadrupole mass spectrometer (Waters Ltd, Herts, UK) with an electrospray ionisation (ESI) source. Separation was performed on an Acquity^TM^ 1.8 µm particle size HSS T3 2.1 × 50 mm column. Prior to analysis, amide parent-to-daughter ion transitions were optimised using MassLynx software with the QuanOptimise application manager. Test compound responses were optimised in positive ion mode by direct infusion in a solvent consisting of 45% 10 mM ammonium formate in deionised water containing 0.1% formic acid and 55% acetonitrile. Eight different cone voltages between 7 and 56 V and five different collision energies between 10 and 50 eV were investigated to maximise analyte response. Amides showing a weak response using the above conditions were optimised in the negative ion mode by direct infusion in a solvent consisting of 45% 10 mM ammonium acetate in deionised water and 55% acetonitrile. Once optimised, multiple reaction monitoring (MRM) was performed to detect the parent amides and amine metabolites in each time point sample. Incubations where the total parent amide decreased below 80% of the t_0_ baseline sample or the amine metabolite was detected were determined to be hydrolysed.

Samples underwent reversed-phase liquid chromatographic (LC) separation using a binary solvent gradient elution profile at a flow rate of 600 µL/min (Table 1). For samples analysed in positive ion mode, mobile phase A comprised of 10 mM ammonium formate with 0.1% formic acid in water and mobile phase B comprised of acetonitrile. For samples analysed in negative ion mode, mobile phase A comprised of 10 mM ammonium acetate in water and mobile phase B comprised of acetonitrile. The injection volume for each sample was 8 µl, with the column maintained at 70 °C.

**Table 1:** Gradient profile for the liquid chromatographic separation samples collected from *in vitro* amide hydrolysis assays. For positive mode ESI mass spectrometric acquisitions, mobile phase A = 10 mM ammonium formate containing 0.1% (v/v) formic acid; mobile phase B = acetonitrile. For negative mode ESI mass spectrometric acquisitions, mobile phase A = 10 mM ammonium acetate; mobile phase B = acetonitrile.

Time (min)	% Mobile Phase A	% Mobile Phase B	Gradient Profile
0.00	100	0	6
0.05	100	0	6
1.00	5	95	5
1.40	100	0	11
1.80	100	0	6

### Data Availability

The datasets generated and analysed during the current study are available from the corresponding author on reasonable request.
